# Pragmatic tolerance: Implications for the acquisition of informativeness and implicature

**DOI:** 10.1016/j.cognition.2011.02.015

**Published:** 2011-07

**Authors:** Napoleon Katsos, Dorothy V.M. Bishop

**Affiliations:** a Research Centre for English and Applied Linguistics, University of Cambridge, UK; b Oxford Study of Children’s Communication Impairments, University of Oxford, UK

**Keywords:** Pragmatics, Acquisition, Informativeness, Implicature, Ambiguity

## Abstract

Recent investigations of the acquisition of scalar implicature report that young children do not reliably reject a sentence with a weak scalar term, e.g. ‘some of the books are red’, when it is used as a description of a situation where a stronger statement is true, e.g. where all the books are red. This is taken as evidence that children do not interpret the sentence with the implicature that the stronger statement does not hold. We propose that (a) these tasks cannot differentiate between actual implicature derivation and mere sensitivity to violations of informativeness; and that (b) children’s apparent failure is not due to lack of competence (whether with informativeness or implicature) but due to their tolerance of pragmatic violations. We report three studies with 5-to-6-year-old English-speaking children and adults employing utterances involving scalar and non-scalar expressions. These show that both age-groups are competent with informativeness, but also tolerant of pragmatic infelicity. These findings have implications for the well-established literature on whether children are aware of ambiguity in referential communication tasks.

## 1 Introduction

The purpose of this paper is twofold. First, we demonstrate that the ability to generate quantity implicatures relies upon competence with informativeness, and that previous investigations of the acquisition of implicature confound these two abilities. Competence with informativeness is also necessary for detecting ambiguity in referential communication tasks. It is therefore not coincidental that recent research on implicatures is converging with well-established research on ambiguity detection with respect to the age at which children reach adult-like competence. Secondly, we challenge the conclusion that children younger than 7 years old lack adult-like competence in these tasks. We show that 5-year-old children are in fact aware of underinformativeness, but that they are also tolerant of pragmatic infelicity, and do not penalise it as strictly as logical falsity. In the most widely-used experimental paradigms, this pragmatic tolerance has led to the misleading conclusion that children are not competent with informativeness. In our first study, we replicate the major finding that children fail with informativeness when a binary judgement task is used. In our second and third studies, we show that young children and adults are sensitive to but tolerant of violations of informativeness. We also show that these findings are not specific to just one type of linguistic expression.

In the next section we briefly discuss quantity implicature, informativeness and ambiguity detection, and highlight the common pragmatic competence that underlies them. We then review research on the acquisition of informativeness and spell out the predictions of our novel account, before verifying these experimentally.

### 1.1 Quantity implicature and informativeness

A fundamental aspect of human communicative competence is the ability to express and infer information beyond what is explicitly said. For example, consider (1) and (2):

**Table:** 

(1)	a.	Mary: Did you dance with John and Bill?
	b.	Jane: I danced with John
	c.	Implicature: Jane did not dance with Bill

(2)	a.	Mary: Did all your class fail the test?
	b.	Jane: Some of my class failed
	c.	Implicature: Not all Jane’s class failed

Given questions (1a) and (2a), Jane can be understood as conveying the literal meaning of (1b) and (2b) as well as (1c) and (2c) respectively, which are not part of what she explicitly said. Grice’s Cooperative Principle and maxims (1975/1989) characterise how such information is communicated. Grice proposed that interlocutors assume each other to be cooperative, and specifically informative, truthful, concise and relevant. If what is explicitly said by the speaker violates any of these assumptions, listeners may infer additional information that would repair such a violation. These pragmatic inferences are known as *implicatures*.

Specifically, the implicature (1c) is derived because Jane is assumed to obey the first maxim of Quantity, which requires her to be as informative as is required for the communicative purpose (Grice, 1975/1989; see also Horn, 1972, 1984; Levinson, 1983; i.a.). The inference would be derived in (at least) two steps. The first step involves determining whether the speaker could have made a more informative statement: in this case, Jane could have said that she danced with John *and* Bill. Given (1a), this extra information would be relevant. The second step involves the negation of the more informative statement that was identified in the first step. This reasoning is valid because, if Jane is adhering to the first maxim of Quantity, she is not being underinformative. Therefore, the most likely reason why she did not make the more informative statement is that it is not true. In this way she communicates the negation of the stronger statement implicitly through a *quantity implicature* (see Geurts (2010), for a detailed discussion).

Similarly, the first step in the derivation of (2c) involves determining that there is a statement (‘all of my class failed’) that would have been relevant and more informative than (2b). In the second step, the hearer reasons that Jane did not make the more informative statement because it does not hold, which is the inference in (2c). Because (2b) is part of a scale of informativeness formed by propositions with the quantifiers ‘some’, ‘many’, ‘most’, ‘all’, it may be considered a special case of quantity implicature, namely a *scalar implicature*.

Investigations of the acquisition of scalar implicature have reported that children younger than 7 years of age cannot derive these implicatures at adult-like levels, or at levels comparable to their competence with explicit meaning (see Barner, Brooks, & Bale, 2011; Feeney, Scrafton, Duckworth, & Handley, 2004; Foppolo, Guasti, & Chierchia, submitted for publication; Guasti et al., 2005; Huang & Snedeker, 2009a; Hurewitz, Papafragou, Gleitman, & Gelman, 2006; Katsos, 2009; Katsos, Andrés Roqueta, Estevan, & Cummins, 2010; Noveck, 2001; Papafragou & Musolino, 2003; Papafragou & Tantalou, 2004; Pouscoulous, Noveck, Politzer, & Bastide, 2007; among others. See Noveck & Reboul, 2009, for an overview).

This is consistent with work on whether children detect ambiguity in referential communication tasks. When children aged 5–6 are given instructions that do not uniquely disambiguate the target referent (e.g. when they are presented with a picture of two men with hats, and told to point to the man with the hat), they still select a referent, and they do not tell the experimenter that s/he did not give them enough information (Ackerman, 1981; Beal & Flavell, 1982; Robinson & Robinson, 1982; Robinson & Whittaker, 1985; among many others; see Plumert, 1996, and Beck, Robinson, & Freeth, 2008, for recent developments and an overview of previous work). Although the research on ambiguity detection has not interacted with that on implicature, both converge on the finding that 5-to-6-year-old children fail to employ the first maxim of Quantity in an adult-like way.

Nevertheless, much younger children succeed with many of the preconditions of pragmatic inferencing, such as attributing and monitoring intentions, tracking their interlocutor’s epistemic state, and counterfactual reasoning (see Clark, 2003; Csibra & Gergely, 2009; Tomasello, 1992; among others). Therefore, the failure of school-age children with implicatures and ambiguity detection is puzzling.

In this paper we investigate why 5-to-6-year-old children fail with informativeness. Our approach has a theoretical and an experimental component. The theoretical part discusses three major points. First, we argue that scalar and non-scalar quantity implicatures are both derived by the same inferential process, and therefore we would not expect one type of implicature to be privileged over the other in acquisition. Second, we show that sensitivity to informativeness is a precondition for implicature derivation, and therefore that informativeness must be considered when interpreting studies that purport to document competence with implicatures (or a lack thereof). Third, we observe that sensitivity to informativeness and the derivation of quantity implicatures are context-dependent and conversational in nature. We conclude that researchers testing pragmatic competence should be aware that participants may be tolerant towards pragmatic infelicity and not penalise it to the same extent as logical contradiction, and should design test materials accordingly.

In the experimental part of the paper, we demonstrate that 5- to 6-year-old English-speaking children are perfectly competent with informativeness, both with scalar and non-scalar expressions. However, they are also tolerant of pragmatic violations. This previously unacknowledged tendency towards *pragmatic tolerance* has significantly masked children’s actual competence with the first maxim of Quantity in a variety of tasks, including the referential communication tasks.

In the following sections we discuss why the type of implicature may be important in the study of acquisition (Section 2.1), the distinction between sensitivity to informativeness and implicature generation (Section 2.2), and why participants may tolerate pragmatic infelicity (Section 2.3).

### 1.2 Scalar vs. non-scalar expressions

With the exceptions of Barner et al. (2011), Papafragou and Tantalou (2004) and Katsos (2009), existing studies on the acquisition of implicature have exclusively considered the scalar type. However, whether these findings should generalise to non-scalar implicatures is a theoretically contested issue. The main difference between cases such as non-scalar (1) and scalar (2) is that, in the former case, the more informative alternative proposition can only be established with reference to context. By contrast, informational scales for expressions such as quantifiers (<some, all>), sentential connectives (<or, and>) and modals (<might, must>) are available without reference to the specific context. Although Grice and subsequent theorists acknowledged this difference, both types of implicature satisfy the criteria to be considered as pragmatic aspects of meaning (see Geurts, 2010; Horn, 1984; Levinson, 1983; Sadock, 1978; for empirical evidence see Breheny, Katsos, and Williams (2006), Katsos (2008), Katsos, Breheny, and Williams (2005), and references therein).

However, recent accounts of implicature differ as to whether these two types of implicature can be treated similarly. *Default* accounts of implicature (e.g. Chierchia, 2004; Levinson, 2000) posit that implicatures arising from context-independent scales are linguistically and psycholinguistically privileged compared to fully context-dependent implicatures. Consequently, children are predicted to acquire the ability to process scalar implicatures earlier than non-scalar implicatures. For instance, Guasti et al. (2005) proposes that the scale <some, all> may form part of the extended lexical entry for ‘some’, thus facilitating the scalar implicature. By contrast, *unitary* accounts of pragmatic inferencing (Carston, 1998; Geurts, 2010; Hirschberg, 1991; Sperber & Wilson, 1986/1995; i.a.) collapse the distinction between scalar and non-scalar implicatures on the grounds that both rely on contextually-specified expectations of informativeness. Preliminary empirical evidence that adjudicates between these two classes of account is available (Papafragou & Tantalou, 2004; see Katsos (2009) for a critical discussion of the methodology), but the issue still remains open to comprehensive experimental investigation.

### 1.3 Sensitivity to informativeness vs. implicature derivation

The most frequently used paradigm for investigating the acquisition of implicature is the binary judgment task (Barner et al., 2011; Feeney et al., 2004; Foppolo et al., submitted for publication; Guasti et al., 2005; Katsos, 2009; Katsos et al., 2010; Noveck, 2001; Papafragou & Musolino, 2003; Papafragou & Tantalou, 2004; Pouscoulous et al., 2007; among others. Many of these tasks are inspired by the Truth Value Judgment Task by Crain & Thornton, 1998). In this task, participants are asked to provide a binary judgment (typically ‘true’/‘false’ or ‘right’/‘wrong’) in cases where a situation is described using a less-than-optimally-informative statement. An example is the scenario in (3), where child participants are told that they are helping ‘Mr. Caveman’, a fictional animated character, to learn English.

**Table:** 

(3)	Scenario: The experimenter, Mr. Caveman, and the participant watch a short animation in which a mouse, who likes vegetables, picks up all of the carrots and none of the pumpkins in the display
a.	Experimenter to Mr. Caveman: What did the mouse pick up?
b.	Mr. Caveman: The mouse picked up some of the carrots
c.	Experimenter to participant: Is that right?

Mr. Caveman’s answer in (3b) is grammatically flawless and logically true, because indeed some of the carrots have been picked up. It is assumed that if participants were to base their response only on what is explicitly said, they should accept Mr. Caveman’s answer. However, if participants interpret Mr. Caveman’s answer with a scalar implicature, to the effect that the mouse did not pick up all of the carrots, they should reject it. Existing studies report that children under 7 years old do not consistently reject underinformative statements of this type, and hence conclude that children do not derive scalar implicatures at adult-like rates. By contrast, children perform at or near adult-like rates with the logical meaning of ‘some’ (e.g. children know that ‘the mouse picked up some of the carrots’ requires that the mouse picked up two or more of the carrots). They also perform at a high level with the meaning of ‘all’ and other quantifiers. Consequently, there is agreement that children are not challenged with quantifier meaning in general, but with scalar implicature specifically.

To the best of our knowledge, studies using the binary judgment task all assume that the participants who reject utterances with a weak scalar term in situations where a strong term is applicable do so because they have derived an implicature. However, as noted by Katsos (2009), this collapses the first and the final step of implicature derivation into a single stage. Katsos (2009) argues that, in these paradigms, the first stage of implicature derivation (awareness that a more informative statement could have been made) suffices to permit the rejection of underinformative utterances. That is, participants could object to underinformative utterances if they recognise that the speaker has given less information than he could, without even considering the implicature arising from the utterance. In the case of (3), participants do not need to calculate the implicature ‘the mouse did not pick up all of the carrots’. Merely recognising that Mr. Caveman only said ‘some of the carrots’ when they witnessed the mouse picking up all of the carrots is sufficient reason to object to the utterance^1^. This applies to non-scalar implicatures as well, as in scenario (4).

**Table:** 

(4)	Scenario: The experimenter, Mr. Caveman, and the participant watch a short animation in which a dog, who is an artist, paints the triangle and the heart in the display but does not paint the star or the square in the display
a.	Experimenter to Mr. Caveman: What did the dog paint?
b.	Mr. Caveman: The dog painted the triangle
c.	Experimenter to participant: Is that right?

Again, simply recognising that Mr. Caveman only said ‘the triangle’, having witnessed the dog painting the triangle *and* the heart, is sufficient reason to object to the utterance, without further requiring the computation of the implicature that the dog did not paint the heart. It is therefore not clear whether binary judgment tasks test participants’ sensitivity to informativeness or their actual derivation of implicatures.

This observation is also potentially critical for other paradigms used to study implicature, including the Felicity Judgment task (Reinhart, 2004; Foppolo et al., submitted for publication; among others), sentence-to-picture matching tasks (Hurewitz et al., 2006) and visual world eye-tracking studies. To take an example of the latter, Huang and Snedeker (2009a, 2009b) investigate whether children aged 5½ and adults use a scalar implicature to select the appropriate referent from a display of four pictures. In an example of their critical condition, two of the pictures are of girls, one of whom has some of the socks (there being other socks in the display), while the other has all of the soccer balls (there being no other soccer balls in the display). Participants are instructed to ‘point to the girl with some of the socks’. The critical issue is whether participants will fixate on the target referent (the girl with some of the socks) before the onset of ‘socks’, which is the semantic disambiguation point. To succeed in this task, we argue that participants do not need to draw an implicature, but simply have to be sensitive to the fact that ‘the girl with some of the…’ would be underinformative if it referred the girl with (all of) the soccer balls. As in the binary judgment paradigm, participants will also succeed in the task if they draw the implicature (‘some but not all of the…’), but once again they do not need to do so.

Sensitivity to informativeness is a precondition for implicature derivation in the Gricean approach and all its major reformulations (e.g. Chierchia, 2004; Geurts, 2010; Levinson, 2000; Sperber & Wilson, 1986/1995; among others). Our interim conclusion is that the literature so far has relied upon paradigms that test the former without necessarily also testing the latter.

### 1.4 Judging underinformative utterances: a case for pragmatic tolerance

The third observation we wish to make is that pragmatic infelicity in the widely used paradigms does not give rise to the same kind of violations as logical falsity. As a result, the pragmatically appropriate response to underinformative utterances in these paradigms is not clear.

First let us suppose that participants are resolving judgement tasks by being sensitive to informativeness (rather than deriving implicatures). Underinformative utterances are strictly speaking true, but sub-optimal. They could therefore be evaluated as better than false utterances but worse than felicitous ones. Hence there may be no single ‘correct’ response for all participants in binary judgement tasks: those who focus on the utterances’ sub-optimality may reject them, while those who focus on the utterances’ truthfulness may accept them.

Now let us suppose instead that participants are actually deriving implicatures. This implicated meaning is *defeasible* or *cancellable*: in other words, it can be revised without giving rise to such strong contradictions as when aspects of explicit logical meaning are revised (see Horn, 1984; Levinson, 1983; i.a.). This intuitive claim is supported empirically (Katsos, 2007: 106ff; Cummins & Katsos, 2010, experiment 3). Participants were presented with short discourses in which an utterance with a scalar expression was followed by an utterance that contradicted either an aspect of the logical meaning of the expression or its scalar implicature. For example, ‘Some of John’s friends are linguists’ was followed either by ‘In fact none of them are’ (logical contradiction) or ‘In fact all of them are’ (pragmatic contradiction). Given a Likert scale, adult speakers of English rated the latter condition significantly more coherent than the former, but less coherent than felicitous controls.

These observations suggest that participants who accept underinformative utterances in binary judgment tasks may do so for either of two radically different reasons. One is that they truly lack some aspect of the necessary competence. The other is that they are fully sensitive to but also tolerant of violations of informativeness. However, both conditions lead to the same behavioural response, namely acceptance of the underinformative utterance. Therefore, it is not possible to disentangle these possibilities using the experimental paradigms discussed so far.

Taking these observations into account, we argue that the interpretation of existing experimental data should be revised, as follows. For paradigms such as the visual world-eye-tracking employed by Huang and Snedeker (2009a, 2009b), correct performance indicates sensitivity to underinformativeness, and perhaps also the ability to derive implicatures. We cannot rule out a scenario in which adults derive full implicatures but children are merely sensitive to informativeness (or, less likely, the reverse). Nor can we rule out differences of this type within age groups.

For binary judgment tasks such as those employed by Noveck (2001), Papafragou and Musolino (2003), Guasti et al. (2005), Barner et al. (2011) and many others, it is again unclear whether the critical competence is sensitivity to informativeness or the ability to derive implicatures. Moreover, the failure to reject underinformative utterances may not indicate a lack of this critical competence, but instead indicate tolerance of pragmatic violations. Again, groups may differ in the reasons for their judgement: it is possible that children’s acceptance of underinformative utterances arises from a lack of competence, while adults’ acceptances are grounded in full pragmatic competence coupled with tolerance of pragmatic infelicity.

Motivated by the observations in these three subsections, we report three studies which aim to clarify the relevant issues. We investigate (a) whether young children’s acceptance of underinformative utterances in binary judgment tasks is due to tolerance of pragmatic violations rather than lack of pragmatic competence; and (b) whether there is a significant difference between their behaviour with scalar and non-scalar expressions.

To do so, we first administer a binary judgment task (experiment 1), which reproduces the finding that 5- to 6-year-old children do not reject underinformative utterances at the rates that they reject logically false ones, or at the same rates as adults. In experiment 2 we administer the same task, but instead of a binary scale (‘right’ or ‘wrong’) we give participants a ternary scale (awarding the fictional character ‘a small’, ‘a big’, or ‘a huge strawberry’). This experiment is the crucial test of our hypothesis on pragmatic tolerance. If children are not sensitive to informativeness, they should give the highest reward for true but underinformative utterances, just as if they were optimal (true and informative). However, under our hypothesis, children are sensitive to underinformativeness but also tolerant of this kind of infelicity. In this case, they should give the middle reward for underinformativeness and reserve the lowest reward for false utterances. In experiment 3, we further test pragmatic tolerance by running a sentence-to-picture matching study with the same materials as experiments 1 and 2.

In interpreting these studies, we are conservative about whether participants are basing their responses on sensitivity to informativeness or actual derivation of a quantity implicature. Specifically, we assume that the former holds, as it is a necessary precondition for the latter. In the General Discussion we explore ways to disentangle these issues. To permit between-task comparisons we use the same experimental stimuli throughout.

## 2 Experiment 1: underinformative utterances in a binary judgment task

This experiment aimed to replicate the typical finding from binary judgment tasks with 5- to 6-year-old children, in which children predominantly accept underinformative utterances.

### 2.1 Method

A computer-based utterance-judgment task was constructed by combining clip art pictures and animations with pre-recorded utterances on Microsoft Power Point software. The task was administered by a single experimenter. At the beginning of the experiment, participants are introduced to a fictional character, Mr. Caveman, who walks to the middle of the computer screen and introduces himself (by means of utterances pre-recorded by a male non-native but proficient speaker of English) and asks participants to help him learn English. The experimenter elaborates that Mr. Caveman knows quite a lot of English, but he would like to learn to speak English perfectly, like the participant does. The experimenter further explains that they will see some stories and that the experimenter will be narrating what is going on in each story. At the end of each story, the experimenter will ask a question and Mr. Caveman will try to answer it. Participants were told that if Mr. Caveman’s answer is right, they should tell Mr. Caveman “that’s right”. If Mr. Caveman’s answer is wrong, they should tell Mr. Caveman “that’s wrong”, and help him by explaining why it was wrong.

In subsequent displays Mr. Caveman is positioned at the bottom of the screen. Each story starts with a screen that is empty except for Mr. Caveman, who asks for the story to begin. Using animations the experimenter introduces the protagonist of each story, the activity that he/she generally likes doing, and the specific options for action available in this story. The protagonist of the story performs some course of action, which is seen in real time (using Microsoft Power Point animation options). For example, in the story where the mouse picks up all of the carrots but none of the pumpkins, there are two piles of vegetables displayed on the left side of the screen, one of five pumpkins and one of five carrots. The mouse moves from the right side of the screen to the pile of carrots and carries each of them back to its starting position, one by one. Each time the mouse comes back with a carrot the experimenter comments ‘Look, he picked up a carrot’. For each story, when the protagonist completes his/her course of action, the experimenter comments ‘and now s/he is very happy’, and then asks Mr. Caveman a question.

### 2.2 Materials

There were 24 items, 12 of which were critical items, testing the ability to reject underinformative utterances. Half of these were for the scalar expression ‘some’, and half for non-scalar expressions, such as the single noun phrase in (4). All the items were answers to an object what-question such as ‘So, what did the mouse pick up?’ or ‘So, what did the dog paint?’ For each of these items Mr. Caveman gives a logically true but pragmatically underinformative response (e.g. ‘The mouse picked up some of the carrots’, ‘The dog painted the triangle’).

There were also 12 stories (six for scalar and six for non-scalar expressions) of similar structure to the critical items. Half of these stories tested whether participants could reject logically false utterances. For example, after witnessing a scenario where a goat jumps over three out of the five fences displayed and over none of the bushes displayed, the experimenter asks ‘So, what did the goat jump over?’ and Mr. Caveman responds ‘The goat jumped over some of the bushes’. The remaining stories tested whether participants could accept optimal utterances (those which are both logically true and pragmatically informative). For example, after witnessing a scenario where the turtle played with three out of the five balls displayed but with none of the trucks displayed, the experimenter asks ‘So, what did the turtle play with?’ and Mr. Caveman responds ‘The turtle played with some of the balls’. Six of the stories testing logical truth and falsity made mention of the weaker term of the scale (‘some’ or single noun phrases) and six mentioned the stronger term of the scale (‘all’ or conjoined noun phrases). See Appendix A for the list of stories and utterances and Appendix B for a sample visual display of a scalar and non-scalar item.

### 2.3 Procedure

The task took between 15 and 25 min to administer and it was part of an experimental session that lasted around 30 min for adult participants and 45 min for children. The session also involved two selection measures for the children, a non-verbal IQ test (Raven’s Coloured Matrices; Raven, Raven, & Court, 1998) and a sentence-repetition task from the NEPSY battery (Korkman, Kirk, & Kemp, 1999). In this and all subsequent experiments reported in this paper, any child falling below 1.25 standard deviations from the age-appropriate mean for the non-verbal IQ test and/or the sentence-repetition task was removed from the sample and replaced. The experiments took place in a relatively quiet room in the children’s school, or at the university for adults.

### 2.4 Participants

The participants were 20 5- to 6-year-old English-speaking children (mean age 5;6, range 5;1–6;2) recruited from primary schools in Cambridge, UK, and 20 adults, students of the University of Cambridge (mean age 23;8, range 20;1–30;3). Two children did not meet the criteria for the selection tasks and were replaced.

#### 2.4.1 Scoring the responses

All the child responses were straightforward ‘yes’, ‘no’, ‘right’ or ‘wrong’ responses, and were scored as correct or incorrect for the critical and control items. All the adult responses to the logically false and the optimal conditions were also ‘yes’, ‘no’, ‘right’ or ‘wrong’. For the underinformative utterances, a range of responses was elicited from the adults, including revisions of the original utterances and meta-linguistic comments. In the main analysis we classified all adult responses that were a straightforward ‘yes’ or ‘right’ as incorrect, on the grounds that the participant did not object to the infelicity. We classified all other responses as correct, regardless of whether the response came as a straightforward rejection, or a more indirect objection, as in any case participants had detected that Mr. Caveman’s utterance was not a perfectly felicitous answer to the question. We also performed a second analysis where we took into account how many of the informative responses came in the form of a straightforward rejection or in an indirect objection.

When participants gave a response other than a straightforward ‘yes’ or ‘right’ and did not spontaneously explain why they gave this response, the experimenter prompted them for an explanation. All participants were able to answer informatively with reference to the appropriate scale e.g. ‘because [the mouse] picked up *all* the carrots’, ‘because [Mr. Caveman] said *some*’. One participant rejected one instance of an item with ‘some’ on the grounds that Mr. Caveman should have used a numeral (he should have said ‘…three of the fences’ rather than ‘…some of the fences’). This response was scored as incorrect. The experimenter then explained that Mr. Caveman does not use number words because he already knows them and he wants to learn other ways of saying things, using words like ‘some’ and ‘all’. After this explanation, the participant did not object again to the use of a quantifier instead of a numeral.

### 2.5 Results: main analysis

Both children and adults were highly competent in the control conditions, rejecting logically false utterances and accepting optimal (logically true and informative) ones at rates over 95%. The only two erroneous responses were elicited from one child rejecting one instance of a scalar expression in an optimal condition (as mentioned above), and another child rejecting one instance of a non-scalar expression in an optimal condition. Turning to responses to the critical underinformative utterances, all the adult responses were rejections or objections. However, the children rejected underinformative utterances at rates of only 29% (26% and 31% for scalar and non-scalar expressions respectively).

Two Mann–Whitney U-tests reveal that the adults performed higher than the children in the underinformative conditions for scalar and non-scalar expressions (both *U *> 4.95, *p* < .001, effect size *r* for non-parametric tests >.78; where >.10 may be considered a small effect, >.30 medium and >.50 large). Within the child group, further pairwise comparisons by Wilcoxon Signed Ranks tests reveal that children performed reliably higher in both the logically false and the optimal conditions compared to the underinformative condition, both for scalars and non-scalars (both *W *> 3.6, *p* < .001, *r* > .8, for false vs. underinformative; both *W *> 3.6, *p* < .001, *r* > .8 for optimal vs. underinformative respectively). Moreover, children’s performance did not significantly differ between scalar and non-scalar expressions in the underinformative condition (*W* = .84, *p* > .1).

Moreover, the rates of rejection of underinformative utterances were reliably above what one would expect if there was no sensitivity to informativeness at all (=no rejections of underinformativeness: One-sample *t*-test, both *t*(19) > 3.1, *p* < .005, effect size *Cohen’s* *d* for parametric tests > .75). Let us also consider participant distribution to examine whether children are uniform in occasionally rejecting underinformative utterances, or whether they cluster in sub-groups. We classified children as consistently underinformative (rejecting 0–1 out of six underinformative utterances) or inconsistent (rejecting 2–4 out of six utterances) or consistently informative (rejecting 5–6 out of six utterances). This classification was done separately for scalar and non-scalars on the grounds that the type of expression might make a difference: for scalar expressions, 13 children were consistently underinformative, 3 were inconsistent, and 4 were consistently informative. For non-scalar expressions, the distribution was 12, 2 and 6 respectively. This classification reveals that the majority (17 and 18 out of 20 children for scalars and non-scalars respectively) were consistent in their behaviour (either informative or underinformative). This finding is in line with the participant distributions reported by Guasti et al. (2005) for children and Bott and Noveck (2004) for adults for the scalar expressions. It further justifies the conclusion that many children lack some aspect of pragmatic competence important to performing this task. Not only was there a difference at the group level between the rejection of underinformative and false utterances, but at the individual level the majority of children (13 out of 20 for scalars and 12 out of 20 for non-scalars) consistently accepted underinformative utterances.

### 2.6 Results: analysis of indirect responses

As mentioned, many adult responses did not consist of a straightforward acceptance or rejection, but were more indirect, phrased as revisions or meta-linguistic remarks. Indirect responses were obtained in the underinformative condition only, at rates of 12% and 33% for scalars and non-scalars respectively (as a proportion of all non-acceptances). More than 90% of these indirect responses were revisions starting with ‘yes’, ‘true’ or ‘right’, followed by the informative description (either with the use of ‘but’ or ‘and’ or without any conjunction). For instance, one adult participant said “yes, he picked up all of them”, and “yes, but he also painted the heart”. The remaining indirect responses did not commit with regard to the correct binary value of the utterance (‘right’ or ‘wrong’) but included explicitly meta-linguistic remarks such as “half right, half wrong”, “I can’t really tell”, “I don’t know”.

If the indirect responses are scored as incorrect, then adult performance in the underinformative conditions falls to 88% for scalars and 67% for non-scalars. Adults are still outperforming the children for both types of expression (Mann–Whitney U: both *U *> 3.03, *p* < .001, *r* > .47), but there is a main effect of expression, with the adults performing higher with scalars than with non-scalars (Wilcoxon Signed Ranks test, *W *= 2.03, *p* < .05, *r* = .45).

The presence of indirect responses in the underinformative but not in the logically false condition indicates that adults do not consider violations of informativeness to be as grave as violations of logical truth. However, no other study using a similar paradigm (e.g. Guasti et al., 2005, experiment 4; Papafragou & Musolino, 2003, both experiments) reports any indirect responses from adults. Could this mean that there is something erroneous with the task that we designed? We think this unlikely on two grounds. First, adults in the studies by Guasti and Foppolo were not given the opportunity to respond orally to the experimenter, but were given the binary choice ‘yes’/‘no’ or ‘right’/‘wrong’ on paper (personal communication). This precludes participants from making the kind of comments that we elicited. Second, excluding indirect responses, we are left with a rate of 88% correct responses to underinformative utterances with scalar expressions, comparable to the 83% reported by Guasti et al. (2005, experiment 4) and the 93% reported by Papafragou and Musolino (2003, experiment 1)^2^. This dispels any concerns that our task elicited fewer categorical rejections from the adults than other tried-and-tested paradigms. Instead, our task design has elicited relevant additional data: even when adults do not categorically reject underinformative utterances, they are not oblivious to pragmatic infelicity, and their responses to underinformative utterances reflect this.

### 2.7 Discussion for experiment 1

Children performed significantly better when the correct response depended exclusively on the logical meaning of scalar and non-scalar expressions than when it also depended on informativeness. In the latter case, but not the former, they also performed worse than the adults. This is exactly the picture documented in previous studies which has been interpreted as evidence that children lack some aspect of pragmatic competence. However, we propose an alternative explanation for children’s acceptance of underinformative utterances, namely that children are tolerant of pragmatic infelicity in binary judgment tasks. To test this claim directly, in the following experiment we give participants a ternary judgment task. If children are not sensitive to violations of informativeness, they should assign the same rating to underinformative and optimal utterances. However, if children are sensitive to informativeness and also tolerant of violations of informativeness they should consistently choose the middle value for underinformative utterances, reserving the highest and lowest value for optimal (true and informative) and false utterances respectively.

## 3 Experiment 2: underinformative utterances in a ternary judgment task

### 3.1 Method

Exactly the same items and scenarios were used as in experiment 1. However, instead of judging whether Mr. Caveman’s response was right or wrong, participants were asked to reward his response using a 3-point scale consisting of different-sized strawberries. These strawberries are introduced as Mr. Caveman’s ‘favourite food’, and are depicted visually in a horizontal line on printed paper, with the smallest on the left and the biggest on the right, each strawberry being twice the size of the previous one. Each point in the scale was explicitly introduced with its label, ‘the small strawberry’, ‘the big strawberry’ and ‘the huge strawberry’. Previous studies in our lab (Katsos & Smith, 2010) using an earlier version of this task revealed that children of this age can give judgements using 5-point Likert-scales, so we did not administer training or special instructions on how to use this 3-point scale.

### 3.2 Participants

The participants were 18 5- to 6-year-old children (mean age 5;8, range 5;3–6;3) attending a primary school in Cambridge, UK and 10 adults (mean age 22;1, range 19;9–25;1). All child participants passed the selection measures.

### 3.3 Results and discussion for experiment 2

The three responses, ‘small’, ‘big’ and ‘huge strawberry’ are coded as response 1, 2 and 3. The adults invariably produced the 3-, 2- and 1-response for the optimal, underinformative and false utterances respectively. The results from the child group are presented in Table 1.

A series of between-group comparisons using Mann–Whitney *U* tests for each cell reveal that children did not perform significantly different than adults in any condition (all *U *< 2.1, *p* > .05).

Within the child group, there were significant differences in the responses to every type of utterance (optimal, underinformative, false) both for both scalar and non-scalar expressions (all six Friedman’s ANOVA *χ*^2^(2) > 20.45, *p* < .001). The preferred responses in the false, underinformative and optimal conditions were 1, 2 and 3 respectively for both expressions (all 12 Wilcoxon Signed Ranks tests *W *> 3.1, *p* < .001, *r* > .73). There was no significant difference between the preferred responses for scalar and non-scalar expressions given the same utterance type (all three *W *< 1.3, *p* > .1). Critically, 2-responses were more frequent in the underinformative than in the false condition, but less frequent than in the optimal condition; 3-responses were more frequent in the optimal than in the other two conditions; and 1-responses were more frequent in the false than in the other two conditions (all *W *> 3.3; *p* < .001, *r* > .77). Thus, at the group level, children were sensitive to informativeness (rating it lower than optimal) but also tolerant (rating it higher than false).

Furthermore, an analysis of individual performance reveals that 16 out of 18 children consistently gave the middle reward to the underinformative utterances (at least 5 out of 6 cases for each expression), with the remaining two children giving underinformative utterances the lowest reward in at least four cases for each expression. Moreover, the children consistently awarded the top reward to the optimal condition and consistently gave the lowest reward to the false condition for each expression (with the exception of one child who did not consistently award the top reward to the optimal condition for scalar expressions).

Thus, given a ternary judgment task, each and every individual child participant revealed consistent sensitivity to underinformativeness (lower reward than optimal) and 16 out of 18 also revealed tolerance (higher reward than false). Every adult participant demonstrated both sensitivity to informativeness and tolerance of pragmatic infelicity.

This has implications for the interpretation of experiment 1, where the majority of children consistently accepted underinformative utterances (13/20 and 12/20 children for scalars and non-scalars respectively). We propose that this group of participants were in fact detecting the violation of informativeness but did not consider it grave enough to warrant the outright rejection of the utterance.

Is it possible that the difference in children’s performance across the two experiments is due to the tasks requiring different types of competence: for example, that experiment 1 requires the derivation of quantity implicatures but experiment 2 only requires sensitivity to informativeness? We cannot see any motivation for postulating this. The experiments do not differ in terms of visual or procedural complexity, and use exactly the same linguistic stimuli, visual animations and overall scenario. Moreover, the experiments do not differ in terms of the meta-linguistic demands of the task, as they both require participants to pass judgment on utterances. The only apparent difference is the use of a ternary scale in experiment 2, which enables participants to give a response that is more lenient than a downright rejection but stricter than a thorough endorsement of the utterance.

If our claims are well-founded, it should follow that children’s pragmatic competence is best investigated using paradigms in which pragmatic tolerance cannot cloud the interpretation of the participants’ performance. To test this supposition, we now turn to the sentence-to-picture matching paradigm, where participants are visually presented with four outcomes of a scenario, and they are asked to select the picture that matches their interpretation of the utterances used in experiments 1 and 2.

## 4 Experiment 3: sentence-to-picture-matching paradigm

### 4.1 Method

The computer-based judgement task used in experiments 1 and 2 was modified as follows. The experimenter explains that participants will see some stories and that Mr. Caveman will narrate what is going on in the story. After being introduced to each story, the participant will be presented with four pictures on the screen, and Mr. Caveman will say what eventually happened in the story that he has in his mind. The participant should then point to the picture that matches Mr. Caveman’s story.

The trials begin as in experiments 1 and 2. After the initial screen display showing the protagonist and the objects that may be affected, participants are shown a second screen divided into four (see Appendix C for a sample visual display). Mr. Caveman then says ‘In my story…’ and then continues his utterance with the pre-recorded utterances used in experiments 1 and 2. Participants are then asked to point to the picture that matches Mr. Caveman’s story.

The pictures differed in the type of objects that were depicted as affected by the protagonist’s actions (e.g. carrots, pumpkins; heart, triangle) and in their quantity (some or all, either or both). For example, in a critical trial for scalar ‘some’, participants were presented with four pictures, corresponding to the situations in which the mouse picked up three out of five carrots, or three out of five pumpkins, or five out of five carrots, or five out of five pumpkins. They then heard ‘In my story, the mouse picked up some of the carrots’. In a critical trial for non-scalar expressions, participants were presented with four pictures, corresponding to the situations in which the dog painted only the triangle, only the heart, the heart and the triangle or the star and the triangle.

### 4.2 Materials

The 24 items used in experiments 1 and 2 were used, modified as described above. The position of the four pictures on the screen was pseudo-randomised. The items were presented to participants in either one of two pseudo-randomised orders.

### 4.3 Procedure

The task took between 15 and 20 min to administer and was part of an experimental session that lasted around 40 min for adult participants and 30 min for children. The session also involved the two verbal and non-verbal IQ selection measures for children. The experiments took place in a relatively quiet room in the children’s school, or at the university for adults.

### 4.4 Participants

The participants were 15 5-year-old English-speaking children (mean age 5;7; range 5;1–6;1), recruited from primary schools in Cambridge, UK, as well as 10 adults, students of various subjects at the University of Cambridge (mean age 23;9; range 19;9–26;3). One child was removed and replaced in the sample on the grounds of low performance in the selection measures.

### 4.5 Results and discussion for experiment 3

Adults performed at ceiling with only one error in a non-scalar condition. The children’s performance was as presented in Table 2.

Between-group comparisons (Mann–Whitney *U*) revealed that children did not perform significantly differently than adults in any condition (all *U *< 2.5, *p* > .05). Focusing on the children, a Friedman’s ANOVA reveals no significant pairwise differences between conditions (*χ*^2^(3) = .84, *p* > .1). This suggests that any difficulty children had was general to all conditions of the task, rather than specific to the conditions contrasting on informativeness. We investigated this further by analysing the children’s erroneous responses for the critical conditions (‘some’ and single noun phrase). The 17% of erroneous responses for ‘some’ were distributed over all the other three pictures on display (7% for the true but underinformative picture, 7% for the picture with the correct quantity but the incorrect object, and 3% for that with the incorrect quantifier and object). A similar pattern arose for the non-scalars (9% errors distributed as 4%, 4%, and 1% for the true but underinformative, false single object, and false two objects respectively).

These findings further document that 5- to 6-year-old children are sensitive to informativeness. Crucially, there is no significant difference between the children’s performance when the selection is based exclusively on logical meaning (for ‘all’ and conjoined noun phrases) and when it is also reliant on informativeness (‘some’ and single noun phrases)^3^.

## 5 General discussion

In this paper we argued from Gricean principles that (a) existing studies of the acquisition of implicature do not unambiguously test whether participants are deriving an implicature or simply exhibiting sensitivity to informativeness; (b) the acceptance of pragmatically infelicitous utterances need not be attributed to lack of pragmatic competence but may instead be due to tolerance of pragmatic violations; and (c) there need not be a difference in the acquisition of pragmatic competence with scalar and non-scalar expressions. We presented three studies documenting that 5- to 6-year-old English-speaking children and adults are indeed both sensitive to and tolerant of violations of informativeness, and that this holds with scalar and non-scalar expressions to the same extent. We argue that this hitherto ignored tendency towards pragmatic tolerance is a potentially significant factor in previous studies that concluded that young children lack some important aspect of pragmatic competence.

We do not deny that other factors proposed in the literature also influence whether participants reject underinformative utterances. Processing demands (Pouscoulous et al., 2007), the presentation of a specific context against which utterances are evaluated (Guasti et al., 2005) and drawing attention to being informative (Papafragou & Musolino, 2003) have been suggested as relevant considerations for children (and the first two for adults as well). Indeed, we would suggest that some of these factors may interact with pragmatic tolerance, e.g. when in a given task it is particularly important to be informative. In this case we might expect participants to treat pragmatic violations as gravely as logical ones. This could include cases of explicit intervention, in which children are trained to correct underinformative descriptions (Papafragou & Musolino, 2003, experiment 2; Guasti et al., 2005, experiment 2) or cases where the question asked highlights a certain contrast, for example if Mr. Caveman were asked ‘Did the mouse pick up all the carrots?’ instead of ‘What did the mouse pick up?’

Turning to the relation between the sensitivity to informativeness and actual implicature derivation, we believe that it is possible to disentangle whether participants are competent with one or the other, but not in judgement tasks or sentence-to-picture-matching paradigms. Implicature derivation can be tapped by paradigms that involve the participant operating on a situation to make it match their interpretation of the critical utterances, rather than evaluating whether the utterances are an adequate description of the given situation. This holds because utterances can be characterised as underinformative only if they are presumed to be describing an existing situation. We are currently exploring this avenue based on the action-based paradigm developed by Pouscoulous et al. (2007, experiment 3).

We do not claim that children’s mastery of informativeness and implicature derivation must develop in tandem. As the former is a prerequisite for the latter, the latter is likely to be psycholinguistically more demanding. Therefore, while it is possible that at least some children in our sample were resolving the tasks by actual implicature derivation rather than mere sensitivity to informativeness, the conservative approach is to interpret child behaviour as driven by the latter. Moreover, children’s early competence in other areas where extensive pragmatic reasoning may be involved, such as word learning, suggests that sensitivity to informativeness may be developed at a younger age (see Clark (2003), Plumert (1996) and references therein). In fact, according to the Gricean approach, we would expect that competence with informativeness is available as soon as the logical meaning of the expressions that form a contrast is acquired.

Furthermore, it is also possible that differences between scalar and non-scalar expressions may appear at some developmental stage, even though these were not evident in 5- to 6-year-old children. An intriguing finding was the difference within the adult group in experiment 1, where more straightforward categorical rejections were elicited for underinformative utterances with scalars than with non-scalars (88% vs. 67%). This could merit further investigation, as it suggests that the difference between expressions may arise later rather than earlier in development, perhaps as the result of repeated exposure to context-independent scales of informativeness.

### 5.1 Final remarks

In the remainder of the general discussion we address two related topics. First, is there other evidence for pragmatic tolerance in the literature and what are the implications for referential communication tasks? Second, why are adults less tolerant than children?

With regard to the first point, several other investigations have inadvertently reported data consistent with pragmatic tolerance. For instance, Paterson, Liversedge, White, Filik, and Jaz (2005) investigated how children and adults understand sentences with ‘only’, such as ‘The woman is only walking a dog’, using sentences without ‘only’ as controls. In their binary judgement task (experiment 1), for conditions where the woman was doing something else as well (e.g. walking a cat), participants rejected the sentences with ‘only’ more than sentences without ‘only’, the difference increasing with age. Since the latter *implies* that the woman is not doing anything else, while the former explicitly states it, this difference is straightforwardly in accordance with the pragmatic tolerance account, where tolerance is restricted to pragmatic rather than semantic infelicity. Moreover, the youngest children (aged 7–8) rejected underinformative utterances at rates of 30% in the binary judgement task. However, in a picture matching task (experiment 2) they selected the picture matching the informative interpretation of the utterance at rates about 85%. This stark contrast can be explained if children in experiment 1 were sensitive to underinformativeness but refrained from categorically rejecting the sentence when given a binary choice. These incidental findings are in line with the pragmatic tolerance account, although the authors do not discuss them in detail.

Further evidence for pragmatic tolerance comes from referential communication investigations, in which children are given underinformative instructions (e.g. when told to point to the man with the hat in the context of two men, each with a hat). An extensive developmental literature investigates whether children are aware of the ambiguity of these instructions (Asher, 1979; Robinson & Robinson, 1976, 1977, 1982; Bearison & Levey, 1977; Ackerman, 1981; Flavell, Speer, Green, & August, 1981; Beal & Flavell, 1982; Robinson & Whittaker, 1985; Plumert, 1996; Beck et al., 2008; among many others). Two of the major findings suggest that they are not. First, children do select a referent in spite of the ambiguity, and, second, they report that the instructions they were given were adequate. The latter is typically investigated by asking the child to tell the experimenter if s/he gave them enough information or not. For example, Robinson and Robinson (1982, experiment 1) report that when asked “Have I told/shown you enough about my card for you to get it right?” (ibid.: 273) 39 out of 52 children aged between 5½ and 7 agree that they have been told enough when in fact the experimenter’s instructions were underinformative. Similar findings are reported in their second experiment, and in several other studies where the question was phrased in terms of a binary choice (Robinson & Whittaker, 1985, experiments 3 and 4; Beal & Flavell, 1982; Flavell et al., 1981, who asked children “Do you think the instructions told you in a good way or in a not-so-good way how to [complete the task]”).

Nevertheless, Beck et al. (2008), Nadig and Sedivy (2002), Nilsen and Graham (2009) and others present evidence that children may be sensitive to the ambiguity in the referential communication task, albeit in more indirect ways. Such evidence has also been available early on in this line of work, as Patterson, Cosgrove, and O’Brien (1980) report that children showed longer reaction times for ambiguous than non-ambiguous messages, and made more eye-contact with the speaker. Plumert (1996) reports that children were delayed in starting to search for an object when the instructions did not disambiguate the hiding place; and Flavell et al. (1981) report that asking children to follow ambiguous instructions to build a model elicited pauses and puzzled expressions. Moreover, Jackson and Jacobs (1982) and Brédart (1984), who used the sentence-to-picture matching paradigm, report that children are very good at selecting the referent for which the instructions would be informative, rather than the referent who was compatible with the instructions but for which the instructions would have been underinformative.

These findings tentatively suggest that children can detect ambiguity, but for some reason resist correcting their experimenter. Stronger evidence to this effect can be adduced from Robinson and Whittaker (1985; experiments 3 and 4) who gave 6-year-old children ambiguous or unambiguous instructions for identifying one of three PlayPeople. After the instructions children were asked two things: first, if they really knew which PlayPerson to select, children were told to point to him/her. But if they did not really know which PlayPerson to select, the children were told to point to a ‘mystery man’. Second, children had to tell the experimenter if s/he had given them enough information to find the PlayPerson or not. Children pointed to the ‘mystery man’ at rates of 68%, showing that in the majority of trials they were aware that they did not know enough to select a PlayPerson. Nevertheless, subsequently they accepted that the experimenter had said enough at rates of 80%.

These findings are straightforwardly in line with our proposal about pragmatic tolerance. Children may choose not to correct their interlocutor when asked to evaluate the instructions in a binary decision task, despite being aware that the instructions are not optimal. Therefore, it is likely that children’s sensitivity to ambiguity in the referential communication task has been underestimated due to pragmatic tolerance^4^.

Additionally, research by Davies and Katsos (2010) using the referential communication paradigm can shed some light on factors affecting the extent of pragmatic tolerance. Motivated by earlier versions of the present work (Katsos & Smith, 2010), Davies and Katsos (2010) tested English-speaking 5- to 6-year-olds and adults with both under- and over-informative instructions. In a binary judgment task, over-informative instructions were accepted at equal rates as the optimal ones by the children, suggesting lack of sensitivity to over-informativeness. The adults on the other hand rejected over-informative instructions significantly more than optimal instructions, giving rise to a similar child–adult discrepancy as in our experiment 1 for underinformativeness. However, when participants were given a magnitude estimation scale, both children and adults rated the over-informative instructions significantly lower than the optimal ones. Thus, Davies and Katsos (2010) conclude that pragmatic tolerance applies to over-informativeness as well. Both children and adults rejected underinformative utterances significantly more often than over-informative utterances in the binary judgement task, suggesting that they are less tolerant of underinformativeness than over-informativeness. This makes sense in the referential communication paradigm, as the underinformativeness of the instructions (e.g. ‘pass me the star’ in a display with two stars) precludes participants from establishing the referent of the noun phrase. Hence, these findings suggest that pragmatic tolerance is further modulated by whether fundamental components of the speech act are jeopardized, such as establishing reference and satisfying presuppositions.

Finally, we consider whether children are more tolerant than adults, and if so, why. In experiment 1, children predominantly accepted the critical utterances, while the adults always objected to them, albeit typically in a more indirect and meta-linguistic way than when rejecting semantically false utterances (around 25% of responses were revisions, hedges, and meta-linguistic remarks). We explore three hypotheses for why children differ from adults.

The simplest explanation is that the difference lies in how children and adults verbalise their judgements. Children may not be as competent as adults in expressing complex judgments such as a ‘yes, but…’ or ‘half right, half wrong’ as opposed to simple ‘yes’ or ‘no’. In this case, young children may default to a simple ‘yes’, and we would expect that the rates of indirect objections will rise along with verbal ability.

Another explanation concerns personality traits that develop over time. On our account, the defeasibility of pragmatic meaning interacts with a decision that must be made at a meta-linguistic level: whether to reject the utterance as worse than optimal, or accept it as better than false. We would expect personality factors such as cognitive flexibility or pedantry to contribute towards the group difference between children and adults, as well as individual differences between participants. Recent research suggests that the prevalence of autistic traits (Nieuwland, Ditman, & Kuperberg, 2010) and participants’ attitudes to honesty and integrity (Bonnefon, Feeney, & Villejoubert, 2009) may affect their response to potentially underinformative stimuli.

A related but distinct explanation concerns children’s certainty about their command of language overall. This could be founded on an experience-based account. Children have less exposure to language than adults, and this limited experience may result in them being less certain about their meta-linguistic judgments, and thus accepting underinformative utterances (while having sufficient experience with truth and falsity to reject semantically false utterances). Indeed, research in the referential communication paradigm and on children’s certainty about their interpretation of ambiguous messages (Robinson & Whittaker, 1985) could inform these hypotheses. These accounts should be empirically testable in future work.

## Figures and Tables

**Table:** 

Starting point	Outcome	Experimenter’s question	Mr. Caveman’s response	Value
*Non-scalar expressions*				
1. The monkey loves eating yummy stuff. There’s a banana, a cake, an orange and a biscuit	The monkey ate the orange and the biscuit	So, what did the monkey eat?	The monkey ate the biscuit	Underinformative
2. The dancer likes packing beautiful clothes in her suitcase. There’s a skirt, a shirt, a pair of shoes and a dress	The dancer packed the shirt and the skirt	So, what did the dancer pack?	The dancer packed the skirt	Underinformative
3. The spaceman wants to buy stuff for his new spaceship. There’s a computer, a desk, a TV and a radio	The spaceman bought the computer and the desk	So, what did the spaceman buy?	The spaceman bought the desk	Underinformative
4. The dog is an artist. He likes painting things. There’s a square, a star, a triangle and a heart	The dog painted the heart and the triangle	So, what did the dog paint?	The dog painted the triangle	Underinformative
5. The flamingo likes giving presents to his friend, the owl. There’s a pen, a ball, a car, and an umbrella	The flamingo gave the owl the ball and the pen	So, what did the flamingo give to the owl?	The flamingo gave the owl the pen	Underinformative
6. The boy likes watering the plants in the garden. There’s a tree, a flower, some grass, and a cactus	The boy watered the flower and the tree	So, what did the boy water?	The boy watered the tree	Underinformative
7. The squirrel is hungry and wants to eat something tasty. There’s a pizza, a hamburger, a lollipop and an ice-cream	The squirrel ate the pizza	So, what did the squirrel eat?	The squirrel ate the pizza	Optimal
8. The doctor likes washing his toys. Here’s a bicycle, a set of drums, a car and a telephone	The doctor washed the car	So, what did the doctor wash?	The doctor washed the bicycle	False
9. Mr. Tough likes pushing things. There’s a box, a car, a desk and a TV	Mr. Tough pushed the TV	So, what did Mr. Tough push?	Mr. Tough pushed the box	False
10. The builder likes carrying things around. There’s a piano, a parcel, a bucket and a ladder	The builder carried the bucket and the ladder	So, what did the builder carry?	The builder carried the bucket and the ladder	Optimal
11. The spaceman wants to play with his toys. There is a puppet, a ball, a truck and a set of pencils	The spaceman played with the truck and the ball	So, what did the spaceman play with?	The spaceman played with the truck and the ball	Optimal
12. The dog wants to eat some fruit. There’s an apple, a pear, an orange and a banana	The dog ate the banana and the apple	So, what did the dog eat?	The dog ate the banana and the orange	False

*Scalar expressions*				
13. The elephant likes pushing things. There are buses and trucks	The elephant pushed all the trucks	So, what did the elephant push?	The elephant pushed some of the trucks	Underinformative
14. The giraffe is hungry for fruit. There are apples and pears on the trees	The giraffe ate all the pears	So, what did the giraffe eat?	The giraffe ate some of the pears	Underinformative
15. Mr. Tough likes lifting stuff up. There are boxes and stones	Mr. Tough lifted all the boxes	So, what did Mr. Tough lift?	Mr. Tough lifted some of the boxes	Underinformative
16. The crocodile wants to play with toys. There are cars and dolls	The crocodile played with all the cars	So, what did the crocodile play with?	The crocodile played with some of the cars	Underinformative
17. The lady likes shooting arrows at things. There are doors and windows	The lady hit all the doors	So, what did the lady hit?	The lady hit some of the doors	Underinformative
18. The mouse wants to pick up vegetables. There are pumpkins and carrots	The mouse picked up all the carrots	So, what did the mouse pick up?	The mouse picked up some of the carrots	Underinformative
19. The goat likes jumping over things. There are fences and bushes	The goat jumped over three out of five fences	So, what did the goat jump over?	The goat jumped over some of the fences	Optimal
20. The dancer likes picking flowers. There are red flowers and yellow flowers	The dancer picked up three out of five red flowers	So, what did the dancer pick up?	The dancer picked up some of the yellow flowers	False
21. The turtle likes playing with his toys. There are balls and trucks	The turtle played with three out of five balls	So, what did the turtles play with?	The turtle played with some of the trucks	False
22. The boy likes lifting things up. There are books and shoes	The boy lifted five out of five books	So, what did the boy lift?	The boy lifted all the books	Optimal
23. The flamingo likes painting things. There are circles and squares	The flamingo painted five out of five circles	So, what did the flamingo paint?	The flamingo painted all the circles	Optimal
24. The postman likes giving yummy stuff to his friend the elephant. There are bananas and biscuits	The postman gave the elephant five out of five bananas	So, what did the postman give the elephant?	The postman gave the elephant all the biscuits	False

**Picture 1:**
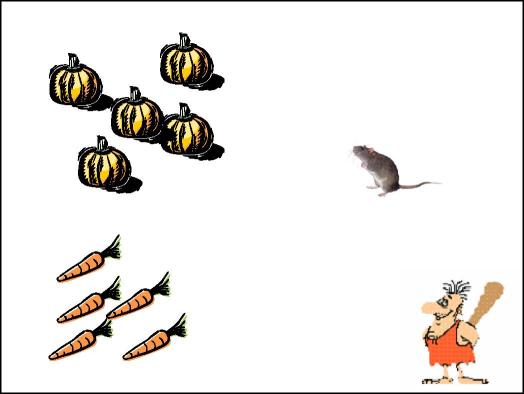
Starting point for scalar.

**Picture 2:**
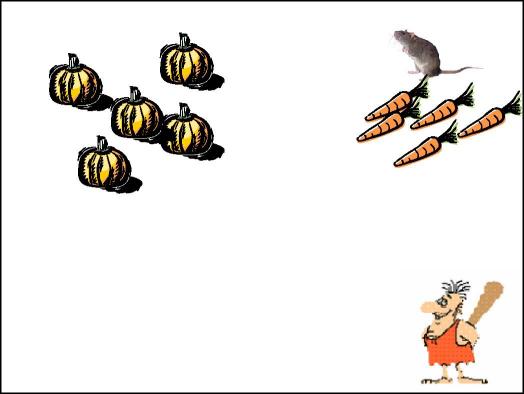
Outcome for scalar.

**Picture 3:**
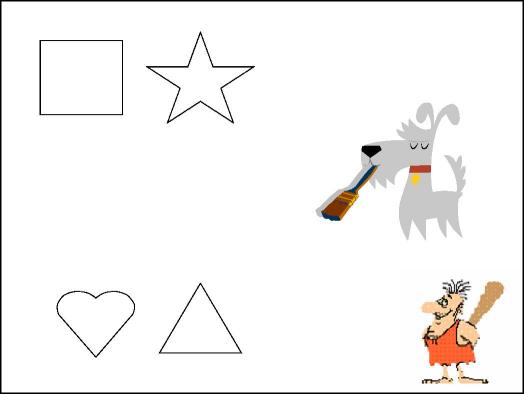
Starting point for non-scalar.

**Picture 4:**
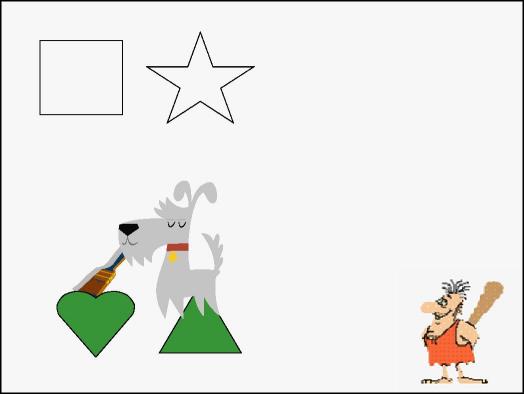
Outcome for non-scalar.

**Picture 5:**
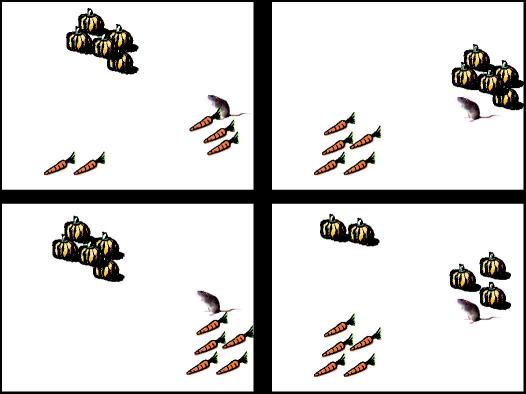
Outcome for scalar.

**Picture 6:**
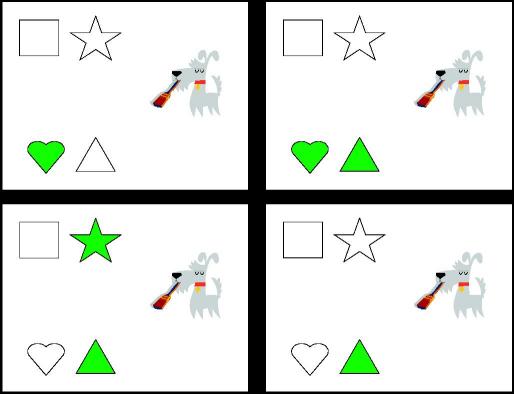
Outcome for non-scalar.

**Table 1:** Proportion of type of response in experiment 2.

Type of utterance	Type of response	Scalar	Non-scalar	Total
Optimal	3 – ‘huge’	85	100	92.5
	2 – ‘big’	0	0	0
	1 – ‘small’	15	0	7.5

Underinformative	3 – ‘huge’	0	6	3
	2 – ‘big’	89	85	87
	1 – ‘small’	11	9	10

False	3 – ‘huge’	5	0	2.5
	2 – ‘big’	0	0	0
	1 – ‘small’	95	100	97.5

**Table 2:** Proportion of correct picture-selection in experiment 3, standard deviation in brackets.

	‘Some’	‘All’	Single noun phrase	Conjoined noun phrase
Children	.83 (.16)	.86 (.15)	.91 (.21)	.89 (.21)

## Appendix A

List of stories and utterances for experiments 1, 2 and 3. The first column describes the starting point of each story, and the second column describes the outcome. The third column presents the question that the experimenter asked. Column four presents the response given by Mr. Caveman. Items 1–12 are the non-scalar expressions, while items 13–24 the scalar ones. The fifth column presents the felicity value of the response, optimal (true and informative), underinformative, or false.

## Appendix B

Sample displays of the starting point and outcome of the scenarios for a scalar (Pictures 1 and 2) and a non-scalar expression (Pictures 3 and 4) in the underinformative condition in experiments 1 and 3.

## Appendix C

Sample displays of the outcome phase of experiment 3 for a scalar and non-scalar expression. The initial state is identical to that of experiment 1 (see Pictures 5 and 6).

## Footnotes

1 Note that the justifications given by participants when rejecting underinformative utterances are straightforwardly consistent with the participants merely being sensitive to underinformativeness (see the justifications quoted in Guasti et al., 2005; Katsos, 2009; Papafragou & Tantalou, 2004; among others). There is no evidence that responses are based on actual implicatures: to the best of our knowledge, no studies report participants objecting on the grounds that ‘some’ means ‘some but not all’.

2 These studies did not include non-scalar items, and hence we could not compare acceptance rates for these items.

3 Note that these findings are not inconsistent with the visual-world eye-tracking study by Huang and Snedeker (2009a) where children did not fixate on the picture consistent with the informative interpretation of the scalar term before the onset of the disambiguating noun. Their results signify that children exhibit a delay rather than a categorical failure to use informativeness, and hence they are consistent with the fact that children perform very well in our study, where children have as much time as they need.

4 Having suggested this, we should also acknowledge that our proposal is orthogonal to other key questions in that literature, such as why children do not refrain from selecting a referent even when they have insufficient information for doing so.
